# A Neuroevolutionary Approach to Controlling Traffic Signals Based on Data from Sensor Network

**DOI:** 10.3390/s19081776

**Published:** 2019-04-13

**Authors:** Marcin Bernas, Bartłomiej Płaczek, Jarosław Smyła

**Affiliations:** 1 Department of Computer Science and Automatics, University of Bielsko-Biała, ul. Willowa 2, 43-309 Bielsko-Biała, Poland; 2 Institute of Computer Science, University of Silesia, Będzińska 39, 41-200 Sosnowiec, Poland; placzek.bartlomiej@gmail.com; 3 Institute of Innovative Technologies EMAG, 40-189 Katowice, Poland; jaroslaw.smyla@ibemag.pl

**Keywords:** traffic signal control, neuroevolution, sensor networks, neural network ensemble, decentralized systems, fuzzy cellular automata

## Abstract

The paper introduces an artificial neural network ensemble for decentralized control of traffic signals based on data from sensor network. According to the decentralized approach, traffic signals at each intersection are controlled independently using real-time data obtained from sensor nodes installed along traffic lanes. In the proposed ensemble, a neural network, which reflects design of signalized intersection, is combined with fully connected neural networks to enable evaluation of signal group priorities. Based on the evaluated priorities, control decisions are taken about switching traffic signals. A neuroevolution strategy is used to optimize configuration of the introduced neural network ensemble. The proposed solution was compared against state-of-the-art decentralized traffic control algorithms during extensive simulation experiments. The experiments confirmed that the proposed solution provides better results in terms of reduced vehicle delay, shorter travel time, and increased average velocity of vehicles.

## 1. Introduction

Properly controlled traffic signals at intersections improve utilization of the existing infrastructure, increase capacity of road network, and alleviate the congestion problem [1]. Real-time monitoring of road traffic parameters is a prerequisite for implementation of effective signal control strategies. The detailed road traffic data can be collected in real-time with use of sensor networks [2]. It should be noted that sensor networks significantly extend the coverage of state-of-the-art traffic monitoring platforms [3]. However, new traffic control algorithms are necessary to effectively utilize the large amount of data delivered from sensor networks.

State-of-the-art traffic control algorithms [4,5] are based on centralized techniques. The centralized traffic control approaches are computationally complex and inherently non-scalable. Low scalability of the centralized control algorithms has motivated the recent interest in decentralized traffic control.

According to the decentralized approach, traffic signals at each intersection in a road network are controlled independently by using an algorithm, which takes control decisions on the basis of real-time traffic data obtained from local measurements. These input data describe current traffic conditions at road segments connected to an intersection.

Thus far, several decentralized traffic control algorithms have been proposed in the literature. The input data of those algorithms consist of parameters that describe the traffic streams passing through an intersection. Output of the algorithm is a control decision that determines which traffic stream (or streams) should get a green signal for a subsequent time interval. The consecutive control decisions are taken in constant time steps. To take control decisions, the decentralized algorithms use various forms of priorities that are assigned to the intersecting traffic streams. The priorities for traffic streams are calculated dynamically based on current traffic parameters. A traffic stream, which gets the green signal, is selected by taking into account the priority levels together with some time constraints that relate to safety and stability requirements.

This paper introduces a decentralized traffic signal control strategy, which can utilize data from sensor network to evaluate priorities of traffic streams and take control decisions. The considered sensor network is composed of nodes installed along traffic lanes. Each sensor node detects presence of vehicle in a section of traffic lane, which is called a cell. The cell states can also be provided from vision based sensors, mobile devices in vehicles or its fusion. In this case, several cells can be monitored by one camera. The detection results are reported by sensor nodes to a traffic lights controller at the nearest intersection.

According to the proposed approach, the priorities of traffic streams are evaluated by the controller with use of an artificial neural network ensemble. The concept of neuroevolution is adapted for training the introduced ensemble of neural networks. During evolution, the performance of the ensemble is evaluated via traffic simulation by using a fuzzy cellular automata model of signalized intersection. The fuzzy cellular automata model facilitates a fast evaluation of vehicle delay at the intersection. The delays of vehicles are considered as fitness function (cost function), which guides the evolution of neural network ensemble towards an optimal solution. Extensive experiments were performed to compare the performance of the proposed method with the decentralized traffic signal control algorithms that are available in the literature.

The paper is organized as follows. Section 2 describes previous works related to the decentralized traffic control and discusses the contribution of this paper. The proposed decentralized traffic control system with neural networks ensemble is presented in Section 3. Section 4 includes experimental results and a comparison of the introduced method with state-of-the-art approaches. Conclusions and future research directions are given in Section 5.

## 2. Related Works and Contribution

In the related literature, several decentralized approaches for traffic signal control have been proposed. These approaches ensure high scalability as they do not require any central controller and do not involve communication between local control units at intersections.

A simple decentralized control algorithm for self-organizing traffic lights (SOTL) is introduced in [6]. According to this algorithm, the priority of traffic stream is calculated by taking into account total waiting time of vehicles and size of vehicle platoons, i.e., groups of vehicles approaching the intersection. Extensions of SOTL algorithm have been proposed to enable utilization of historical traffic measurements and data delivered from existing vehicle detectors.

Lammer and Helbing [7] introduced a decentralized traffic control algorithm, which takes into account traffic stream priorities that correspond to expected increase of vehicle delay. The future delays of incoming vehicles are estimated in this method via short-term traffic flow prediction by using a fluid-dynamic model of traffic flow. In [8], this method is extended by introducing a decentralized traffic signal system, which utilizes an interval microscopic traffic model to predict delays of individual vehicles in a short time horizon and evaluate uncertainty of this prediction.

Real-world experiments reported in [9] confirm the high effectiveness of the above decentralized control algorithms. It is shown that the decentralized methods can provide decreased vehicle delays and increased network capacity in comparison with popular adaptive traffic control systems.

Back-pressure [10] is a decentralized signal control method, which introduces so-called pressures corresponding to lengths of vehicle queues. Initially, the back-pressure algorithm was intended for routing in wireless networks to provide maximum throughput under the assumptions that all links in the network have infinite capacities. This concept was then adapted to urban road networks for signal control. According to the back-pressure algorithm, a higher priority is assigned to traffic streams with high upstream pressure and low downstream pressure. The priority is proportional to difference of queue lengths in inbound and outbound traffic lanes at the intersection.

Another decentralized control strategy for traffic signals is based on so-called virtual impulses that reflect the impact of signals and preceding cars on desired vehicle speed [11]. This approach was inspired by the impulse definition from physics. The virtual impulses are calculated by taking into account a reduction of vehicle speed caused by the preceding cars or red signals. An optimal velocity model is used to predict the virtual impulses for different signal switching scenarios. The traffic signals are controlled according to the signal switching scenario, for which the predicted virtual impulse is minimum. Thus, the virtual impulse for a given traffic stream can be interpreted as its priority level.

In [12], the Ising model of ferromagnetism is used to describe chaotic behavior of decentralized traffic signals. States of the traffic signals are represented by atomic spins on a two-dimensional lattice. A simple traffic control algorithm is proposed, in which priorities of traffic streams correspond to numbers of vehicles approaching the intersection. The traffic signals at intersection switches if the difference between numbers of vehicles approaching red signal and vehicles approaching green signal is above a predetermined threshold.

In [13], a decentralized algorithm is proposed, which controls traffic signals at intersection by using a set of control rules. The control rules take into account aggregated parameters of intersecting traffic streams, i.e., number of vehicles, average vehicle position, and range of vehicle position. Optimization of the control rules is performed with application of a self-adaptive evolutionary strategy. Fitness function for the evolutionary strategy is evaluated via traffic simulation in a cellular automata model.

A multiagent system for distributed traffic signal control is proposed in [14]. Each agent in that system consists of a five-layer fuzzy neural network and is responsible for controlling traffic signal at one intersection in the road network. Learning of the agent is divided into three stages that involve reinforcement learning, weight adjustment, and adjustment of fuzzy relations with application of evolutionary algorithm. The traffic parameters are sampled by agents every 10 s. Each agent has a set of several signal plans that can be used to control the traffic signals at intersection. The task of the agent is to select an appropriate signal plan based on its own perception of the traffic state at its intersection.

Day and Bullock [15] investigated the impact of signal phases for pedestrian on performance of the distributed traffic signal control. They compared the performance of distributed control algorithm with actuated coordinated control. The experimental results show that large reductions of total delay can be achieved in some of the considered scenarios. The distributed signal control is found to be more flexible than the coordinated one. The authors concluded that their results are promising for future development of the distributed algorithm.

In [16], it is noted that the new distributed traffic control systems require advanced sensors that increase the installation cost and can hinder their deployment. To solve this problem, the authors introduced an approach that utilizes simple sensors to detect the presence of vehicles in a few selected locations. Results of vehicle detection are used to predict arrivals and departures at the controlled intersection. The predictions are obtained in that method via simulation of vehicle movement. Results reported in that work show that the introduced approach decreases installation costs and increases robustness of the distributed traffic control system against sensor failures.

Recently, a distributed traffic control system is proposed, which is based on so-called digital infochemicals [17]. The concept of digital infochemicals mimics the natural chemical substances that transmit information generated by one organism to a second organism in the environment. In the distributed traffic control system, the digital infochemicals refer to information generated by vehicles and dissipated by the urban transportation infrastructure. The authors demonstrated that their distributed control strategy performs significantly better than the state-of-the-art solutions in a small case study.

Nilsson and Como [18] introduced a fully decentralized traffic signal control strategy with variable cycle length. An advantage of this strategy is that it does not require any information about the structure of road network or the rates of turning vehicles at intersections. The aforementioned control strategy is based on a well-known rule in traffic engineering, which prescribes that during periods of higher demand, it is convenient to have longer signal cycles. They compared the performance of the introduced strategy with variable cycle length in a simulation environment to a similar strategy with fixed cycle length and with the traditional fixed-time control method. The results show that variable cycle length significantly reduces the overall queue lengths of vehicles in the road network for a wide range of traffic volumes.

A distributed traffic control strategy based on cell-transmission model of road traffic is presented in [19]. The authors proposed a simplified traffic model to eliminate the drawbacks of the state-of-the-art cell-transmission model in describing the evolution of traffic flow on the signalized intersections. They designed a set of control rules using the subgradient descent method to update the status of traffic signals at intersections. The proposed approach allows the traffic signal timing plan to be found in a fully distributed manner. Results of simulation reveal benefits of the proposed method in comparison to the mixed-integer linear programming approach.

In this study, the performance of a distributed system was analyzed, which controls the traffic signals at intersections by using ensemble of neural networks. According to the authors’ knowledge, this is the first study focused on designing a neural network ensemble to improve the performance of distributed, self-organizing traffic control in a road network with multiple signalized intersections. The experiments reported in this paper involved a comparison of the traffic control performance achieved for various neural network topologies. Based on the experiments, an ensemble of neural networks with new topology is presented, which can be effectively utilized for making control decisions by the decentralized traffic signal systems. A neuroevolution strategy [20] is also explored in this paper to enable effective training of the neural network ensemble. Effectiveness of the neuroevolution approach was compared for different implementations that utilize various metaheuristics [21], i.e., genetic algorithm, evolutionary strategies, and particle swarm optimization. Results of experimental evaluation reveal that the proposed solution achieved reduced vehicle delay, shorter travel time, and increased average velocity in comparison with the state-of-the-art decentralized traffic control algorithms.

A new topology of neural network is proposed for aggregation of traffic data collected by sensor network. This topology reflects the design of the considered intersection. It means that the neurons and their connections correspond to configuration of traffic lanes, possible movements at the intersection and signal groups, i.e., predefined subsets of traffic streams that are controlled by identical traffic signal indications. This neural network enables adaptive aggregation of input data that describe counts of vehicles. Therefore, the aggregation scheme can be optimized during training of the neural network. For instance, the weights in neural network can be adjusted to determine that a vehicle close to the intersection has a greater impact on the control decision than a vehicle for which the distance to the intersection is large. It should be noted that the existing decentralized traffic control methods assume that the input data are aggregated in advance and the same predetermined aggregation scheme is used for all intersections.

To evaluate the priorities of intersecting traffic streams, the proposed method uses a set of fully-connected neural networks (FCNNs) [22]. The application of FCNNs allows the number of neurons and inter-neuron connections to be reduced in comparison with the popular multilayer architecture of traditional neural networks. Thus, the training of the proposed ensemble can be accomplished in shorter time.

The proposed neural network ensemble ensures fast evaluation of the priorities for all signal groups and allows the control decision to be made in a short time interval. Thus, the traffic signals can rapidly respond to changing traffic situations. In the case of the state-of-the-art methods that utilize traffic models to predict future traffic evolution, the decision-making procedure requires long time especially for complex intersections, where many possible combinations of traffic lanes have to be considered when deciding which lanes should get green light.

## 3. Proposed Neural Network Ensemble

**Figure 1:**
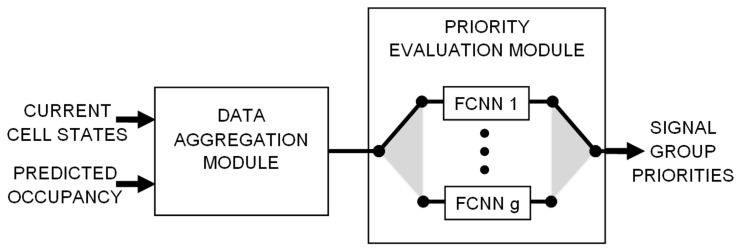
Structure of the neural network ensemble.

A general structure of the proposed neural network ensemble is presented in Figure 1. The main components of this structure are the data aggregation module and the priority evaluation module. Detailed information about these modules is provided in the following subsections.

Input data of the proposed ensemble include detailed information from sensor network about vehicles that are present in road segments connected to an intersection as well as predicted traffic parameters (predicted occupancy of traffic lanes). Based on these data, the ensemble evaluates priorities of traffic streams.

**Figure 2:**
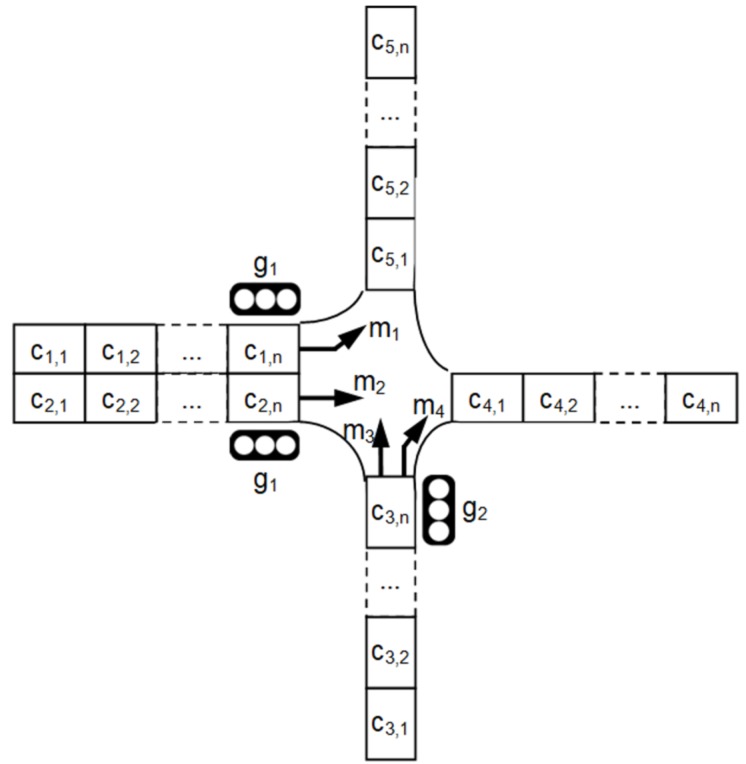
Example of signalized intersection.

The input data collected by sensor network describe current states of so-called cells, i.e., traffic lane segments of equal length. Sensor nodes detect presence of vehicles in particular cells. A cell can be empty or occupied by a vehicle. These two states are defined as binary variables: $c_{i,j}=0$ denotes empty cell and $c_{i,j}=1$ corresponds to occupied cell (*i* and *j* are indices of the traffic lane and the cell, respectively). An example of intersection with traffic lanes divided into cells ($c_{i,j}$) is shown in Figure 2. The occupancy of traffic lane ($d_{i}$) is defined as the ratio of the number of occupied cells to the total number of cells in a given lane (*i*). Output variables of the proposed ensemble are priorities of signal groups.

The parameters (cell states and predicted occupancy of traffic lanes) were selected based on the results available in the related works, where it was demonstrated that a representation of traffic streams by cellular automaton model is suitable for applications in distributed control of traffic signals at intersections [6,13]. The cell state informs us if the shortest considered section of traffic lane (cell) is currently occupied by a vehicle. In contrast, the predicted occupancy denotes the fraction of cells in traffic lane that will be occupied at the next time step of the control procedure.

### 3.1. Data Aggregation Module

The basic approach in state-of-the-art methods that collect data from sensor networks is to calculate the priority for a given signal by taking into account the sum on occupied cells (number of vehicles) in a traffic lane controlled by the considered signal. In contrast, the proposed data aggregation module calculates the priorities as weighted sums of the occupancy, where the weights are adjusted during training of the neural network.

**Figure 3:**
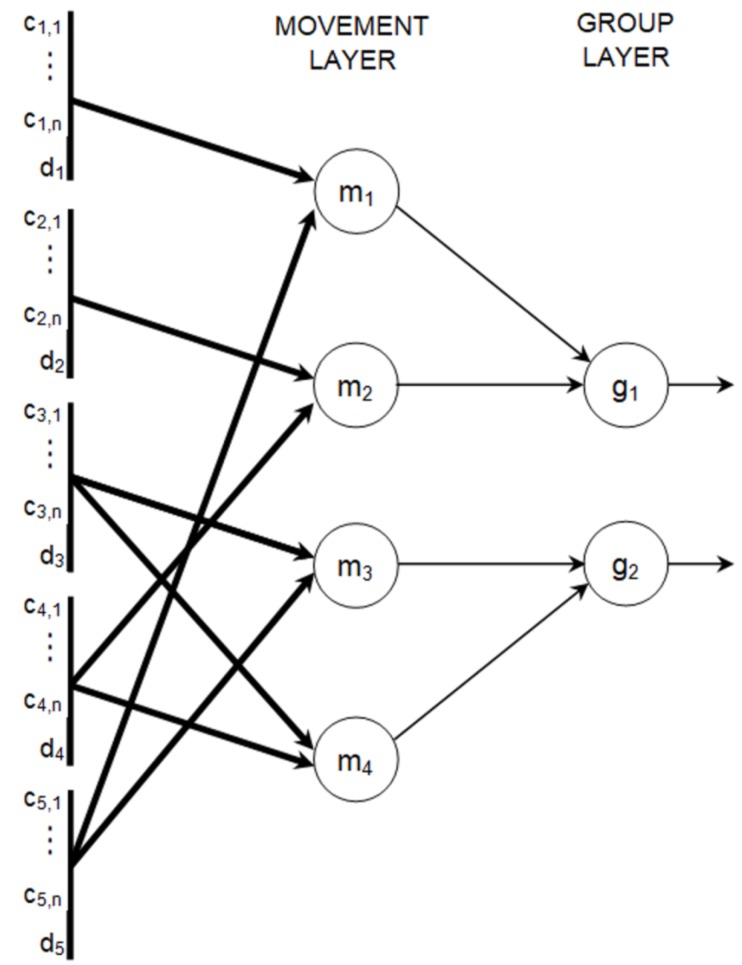
Topology of the data aggregation module for the intersection in Figure 2.

The data aggregation module includes a neural network with three layers (input layer, movement layer and group layer). Topology of this neural network is determined by the design of the intersection. Figure 3 shows construction of the data aggregation module for the intersection presented Figure 2.

Formally, the signalized intersection is represented as a triple (L,M,G), where *L* is set of traffic lanes, *M* denotes set of movements, and *G* is set of signal groups. As mentioned above, each traffic lane (*l*) is divided into cells: $l_{i}=\left\{c_{i,1},c_{i,2},...,c_{i,n}\right\}$. The movements (*m*) are defined by pairs ($l_{a},l_{b})\in L^{2}$, such that a vehicle may enter the intersection through lane $l_{a}$ and exit through lane $l_{b}$. Signal group (g⊂L) is a set of inbound traffic lanes that are controlled by identical traffic signal indications, i.e., simultaneously gain and lose the right-of-way. For example, for the intersection shown in Figure 2, the following representation is used: $L=\{l_{1},l_{2},...,l_{5}\},$ $M=\{m_{1},m_{2},...,m_{4}\},$ $m_{1}=\left(l_{1},l_{5}\right),$ $m_{2}=\left(l_{2},l_{4}\right),$ $m_{3}=\left(l_{3},l_{5}\right),$ $m_{4}=\left(l_{3},l_{4}\right),$ $G=\{g_{1},g_{2}\},$ $g_{1}=\left\{l_{1},l_{2}\right\},$ $g_{2}=\left\{l_{3}\right\}.$

Input layer of the data aggregation module consists of neurons that correspond to the parameters describing traffic situation for each lane, i.e., cell states ($c_{i,j}$) and predicted occupancy ($d_{i}$). Neurons in the input layer are passive, which means that they just relay the values from input to outputs without modification. The number of these neurons equals (n+1)·#L, where *n* is the number of cells in single traffic lane and # denotes cardinality of the set. Collections of the neurons in input layer that correspond to particular traffic lanes are represented in Figure 3 by thick vertical lines.

Each neuron in the movement layer reflects a possible movement (*m*) of cars from inbound traffic lane ($l_{a}$) to outbound traffic lane ($l_{b}$) (examples of movements are shown by arrows in Figure 2). Inputs of neuron *m* in the movement layer are connected to outputs of neurons $c_{i,1},c_{i,2},...,c_{i,n}$ and $d_{i}$ in the input layer, where $i\in \{l_{a},l_{b}\}$. These connections are presented in Figure 3 by thick arrows. It should be noted that each thick arrow in Figure 3 represents multiple inter-neuron connections, and each individual inter-neuron connection is associated with a synaptic weight.

The number of neurons in group layer is equal to the number of signal groups at the intersection. Let us consider the movement $m=(l_{a},l_{b})$. Output of neuron *m* in the movement layer is connected to input of neuron *g* in the group layer if the inbound traffic lane $l_{a}$ of movement *m* belongs to the signal group *g*. Such inter-neuron connections are shown in Figure 3 by the fine arrows. All neurons in the aggregation module have linear activation function.

### 3.2. Priority Evaluation Module

Single output of the data aggregation module can be interpreted as aggregated (weighted) occupancy of the traffic lanes that are controlled by one signal group. Such outputs are fed into the priority evaluation module, which includes one FCNN for each signal group. The number of FCNNs implemented in this module equals #G. Let $FCNN_{g}$ denote the neural network assigned to signal group *g*. $FCNN_{g}$ is selected to be used for evaluating the priorities of all signal groups when *g* is the active group, i.e., displays green signal.

The FCNN is implemented in the proposed ensemble to represent dependency between the occupancies aggregated for signal groups and priorities of these groups. It should be noted that priority of a given signal group depends not only on the occupancy of traffic lanes controlled by this particular group but also on the occupancies of the remaining traffic lanes. FCNN reproduce the aforementioned dependency between #G aggregated occupancies and #G priorities of signal groups.

**Figure 4:**
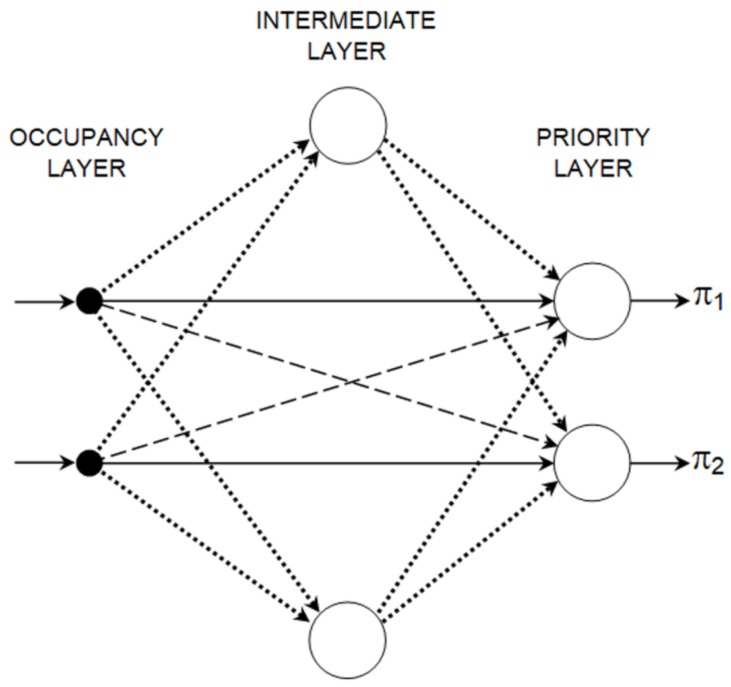
Topology example of FCNN in priority evaluation module.

It should be noted here that the FCNN topology was selected for application in the proposed ensemble based on results of previous works, which have shown that this topology allows the number of neurons (and synaptic weights) to be reduced in comparison with the multilayer perceptron topology [23,24]. The lower number of neurons and synaptic weights is important for increasing the speed of neuroevolution. An example of the FCNN [25] for the proposed priority evaluation module is shown in Figure 4. This example concerns an intersection with two signal groups.

During neuroevolution, a weight value is selected from a predetermined range for each connection between neurons in the FCNN. The allowed range of weight values for inter-neural connections in the proposed FCNN depends on the type of the connection. In the case of connections between the occupancy layer and the priority layer, the weight range is (0, 1] provided that both connected neurons are assigned to the same signal group (solid lines in Figure 4) and the weight range is [−1, 0) if the connected neurons represent different signal groups (dashed lines in Figure 4). The reason behind these constraints is that a higher occupancy of traffic lanes controlled by a given signal group increases priority of this signal group and reduces the priorities for the remaining signal groups. For the connections with neurons in intermediate layer (dotted lines in Figure 4), the weights can take values from range [−1, 1]. The priority layer neurons have tan-sigmoid activation function, and the intermediate layer neurons have linear activation function.

### 3.3. Decentralized Traffic Control Algorithm

The proposed neural network ensemble is used to estimate priorities (π) of all signal groups (g∈G) for the decentralized traffic control algorithm (Algorithm 1). This algorithm is executed in constant time steps of 1 s. Under normal operation, the signal group with the highest priority is set to be the active group (g*) that should indicate green signal. In the case when two or more signal groups obtain the same priority, the group that has been inactive for the longest time is selected to be active. If the active group is changed, then a setup time (consisting of inter-green period and minimum green period) has to be introduced due to safety requirements. During setup time, the priority estimation is skipped because the active group cannot be changed. Moreover, the algorithm assumes that each signal group should be activated at least once during period of *T* time steps. Thus, a signal group, which was inactive for more than T-1 time steps is activated immediately, regardless of the priorities. Decentralized traffic control algorithm (Algorithm 1), for each time step, is defined as follows:

**Algorithm 1:** Decentralized Traffic Control Algorithm
  acquire current cell states $c_{i,j}$  predict occupancy of traffic lanes $d_{i}$  if not setup time then    begin // minimum green period constraint    for each signal group g∈G do    if signal group *g* was not active longer than T-1 time steps then     begin //inter-green period constraint     active group g*:=g     go to 13     end    estimate priorities $\pi_{g}$ for all signal groups *g* = 1, ..., #G    active group g* := control group with the highest priority $\pi_{g*}=max\left\{\pi_{1},...,\pi_{\#G}\right\}$    end


Algorithm 1 uses the trained neural network ensemble to estimate priorities for all signal groups (Line 11). To train the proposed ensemble, the neuroevolution strategy described in following section was proposed.

### 3.4. Neuroevolution Strategy

A neuroevolution approach was included in the proposed system to train the introduced neural network ensemble. The aim of the neuroevolution is to optimize the connection weights in both the aggregation and the priority evaluation module. Moreover, the neuroevolution procedure optimizes the number of neurons in the priority evaluation module.

**Figure 5:**
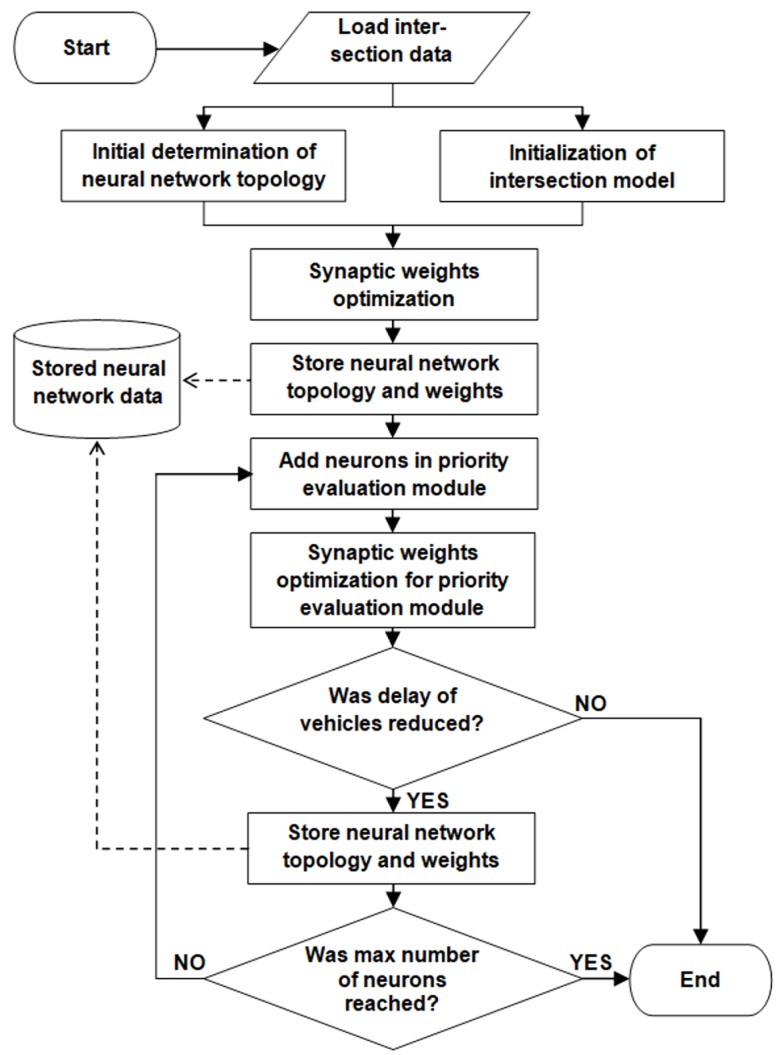
Overview of the neuroevolution strategy.

An overview of the proposed neuroevolution strategy is presented by the flow chart in Figure 5. It should be noted that this strategy can be applied independently for each signalized intersection in the road network. Output of the neuroevolution strategy includes the stored neural network topology and weights. These neural network data are used by traffic control algorithm (Algorithm 1) to evaluate priorities of signal groups.

At the initial stage of neuroevolution, the data describing design of the considered intersection (lanes, movements, and signal groups) are utilized to determine the neural network topology as well as to configure the intersection model. The initial topology of neural network ensemble is determined according to the assumptions discussed in Section 3.1 and Section 3.2. Additionally, the number of neurons in the intermediate layer is set to zero for all FCNNs contained by the priority evaluation module.

The intersection model is necessary in the proposed strategy for evaluation of an objective function during optimization of the synaptic weights. Details of this model, which is based on fuzzy cellular automata, are presented in Section 3.4.1. Initialization of this model includes creating data structures for cells in particular traffic lanes and setting parameters that determine which traffic lanes get green light if a given signal group is activated.

After the initial topology of the neural network ensemble is determined, optimization of synaptic weights is performed for both the data aggregation module and the priority evaluation module. In this study, different soft computing methods were applied for optimization of the synaptic weights in neural network ensemble. The soft computing methods that were considered include genetic algorithm, evolutionary strategies and particle swarm optimization algorithms. The fuzzy cellular automata model was used for all aforementioned optimization approaches to evaluate the objective function (fitness function), which is defined as mean delay experienced by a vehicle at the intersection.

To reduce the amount of weights that have to be optimized, it was assumed that the connections between input neurons and movement neurons in the data aggregation module have the same weights:

(1) $$\begin{array}{c} weight\left(c_{a,1},m_{x}\right)=weight\left(c_{b,1},m_{y}\right),\\ weight\left(c_{a,2},m_{x}\right)=weight\left(c_{b,2},m_{y}\right),\\ ...\\ weight\left(c_{a,n},m_{x}\right)=weight\left(c_{b,n},m_{y}\right),\\ weight\left(d_{a},m_{x}\right)=weight\left(d_{b},m_{y}\right), \end{array}$$

if traffic lanes $l_{a}$ and $l_{b}$ belong to the road(s) of the same category and direction (inbound or outbound). The notation weight(α,ω) is used to represent the synaptic weight assigned to connection between neurons α and ω. It should be noted here that the above assumption also applies if lanes $l_{a}$ and $l_{b}$ are elements of the same movement, i.e., if a=b. During preliminary experiments, it was confirmed that this assumption improves quality of results obtained from the optimization algorithms based on soft computing methods. The road categories are determined regarding speed limit and road type (arterial/main road or minor/side road).

The initial topology of neural network is stored in the memory together with the optimized synaptic weights. At the next step, topology modifications are introduced in the priority evaluation module. During single iteration of this procedure, one neuron is added in the intermediate layer of each FCNN and the optimization of synaptic weights for the priority evaluation module is executed. It should be noted that weights in the data aggregation module remain unchanged. If current modification provides a lower delay of vehicles than the previously stored configuration of the neural network ensemble, then the modified topology and weights replace the stored ones. The modification procedure ends if the current delay is not lower than the result obtained for previously stored (simpler) neural network or if the maximum number of neurons in intermediate layer is reached.

The final result of the neuroevolution includes the last stored topology and synaptic weights of the neural network ensemble.

#### 3.4.1. Simulation-Based Evaluation of Objective Function

The proposed neuroevolution strategy requires the objective function to be evaluated for a large set of candidate solutions in a number of iterations. In the proposed approach, the objective function is evaluated via simulation. A fuzzy cellular automata model [26] is used to simulate the signalized intersection as it speeds up the simulation process in comparison to other simulators. The cellular automata model provides simple representation of the intersection using discrete variables instead of continuous ones. The length of a cell (as presented in Figure 2) is equal to 7.5 m. Thus, the model has low computational complexity and ensures appropriate level of details. The application of cellular automata allows us to execute the evolutionary strategy within acceptable time limits without using expensive high-performance computers.

Furthermore, locations and speeds of individual vehicles are described by triangular fuzzy numbers. Location refers to cell occupied by a vehicle. Speed is expressed in cells per time step of the simulation (1 s). The locations and speeds of all vehicles in this model are updated at each time step of the traffic simulation according to rules of the cellular automata.

The fuzzy cellular automata allow the objective function to be evaluated in a single simulation run and in the same time estimate the uncertainty of simulation results. This approach eliminates the necessity of time-consuming Monte Carlo simulations that use probabilistic models. The Monte Carlo method would require repeating the traffic simulation multiple times in order to reproduce the uncertainty related to random driver behavior. In contrast, the fuzzy cellular automata incorporate many potential scenarios (driving styles) in a single simulation.

**Figure 6:**
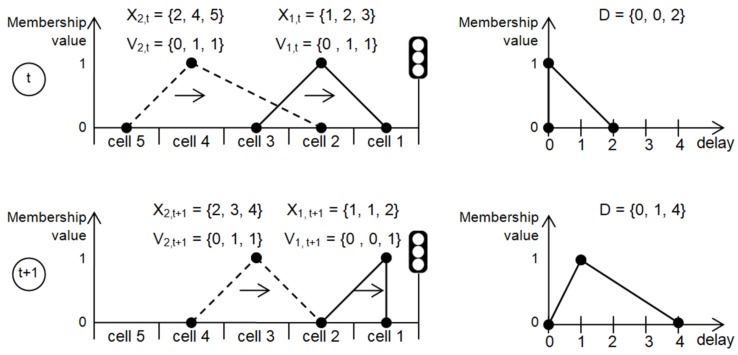
Evaluation of vehicles delay in traffic model based on fuzzy cellular automata.

Figure 6 shows an example of the fuzzy cellular automata model representing single traffic lane with two vehicles approaching red signal at an intersection. In this example, the first vehicle has to stop in Cell 1 due to the red signal. Consequently, the second vehicle will stop in Cell 2. Traffic direction is indicated by the horizontal arrows in the left part of Figure 6. The triangular fuzzy numbers $X_{i,t}$, $V_{i,t}$ describe location and speed of *i*th vehicle at time step *t*. The charts in right part of Figure 6 present triangular fuzzy numbers that describe total delay of the simulated vehicles. Each triangular fuzzy number *F* is defined by three real numbers {l(F),m(F),u(F)}. The components l(F), m(F) and u(F) denote, respectively, the smallest possible value, the most possible value, and the largest possible value.

During traffic simulation, different cellular automata rules are applied to update components (*l*, *m*, *u*) of the fuzzy numbers representing vehicle speed $V_{i,t}$ and position $X_{i,t}$. The components $l(V_{i,t})$ and $l(X_{i,t})$ are determined by an update rule, which reflects a calm driving style. Another rule that corresponds to aggressive style of driving is used for calculation of the components $u(V_{i,t})$ and $u(X_{i,t})$. Finally, the most possible location and speed (components $m(V_{i,t})$ and $m(X_{i,t}))$ are updated with use of a cellular automata rule which simulates behavior of an average driver. Detailed definitions of the cellular automata rules that are applied in this study can be found in [26].

The total stop delay of vehicles is determined as triangular fuzzy number *D* based on the speed values $V_{i,t}$. The component l(D) is incremented at time step *t* if $l(V_{i,t})=0$. Similarly, m(D) is incremented if $m(V_{i,t})=0$, and u(D) is incremented when $u(V_{i,t})=0$. It should be noted that the update of the total delay *D* is performed at each time step of the simulation by taking into account all vehicles (*i*). After finishing simulation, the three components of *D* are divided by number of simulated vehicles to obtain the average vehicle delay.

#### 3.4.2. Optimization of Synaptic Weights

As mentioned above, the synaptic weights for the proposed neural network ensemble are optimized by means of the population-based metaheuristic methods. In this study, three different metaheuristic optimization algorithms were applied during the experiments: genetic algorithm (GA) [27], evolution strategy (ES) [28] and particle swarm optimization algorithm (PSO) [29].

For all of the considered metaheuristics, the candidate solutions play the role of individuals in a population (genomes or particles). These solutions are represented by vectors of the real-valued weights. The weights are subject to predefined lower and upper bounds. Quality of the candidate solutions is determined by objective (cost) function, which has to be minimized. The objective function for a given candidate solution is evaluated as mean stop delay experienced by vehicles at a signalized intersection, where the traffic signals are controlled according to the priorities calculated by the neural network ensemble with the synaptic weights determined by the given solution. The value of the objective function is calculated in a road traffic simulation by using an intersection model, which is based on fuzzy cellular automata. This model allows the mean vehicle delay to be evaluated in form of a fuzzy number, as discussed in Section 3.4.1. Selection of the best candidate solutions is based on comparison of the fuzzy numbers that describe objective function values. The probabilistic approach to fuzzy numbers ordering [30] was used in this study to compare the quality of candidate solutions.

To explain how the fuzzy numbers are compared, let us assume that *A* and *B* are some arbitrary fuzzy numbers with the membership functions given by $\mu_{A}\left(x\right)$ and $\mu_{B}\left(x\right)$, respectively. The probabilistic approach to fuzzy numbers ordering allows us to calculate the probability P(A<B) that condition A<B is satisfied. To this end, the following α-cuts are analyzed: $A_{\alpha }=\left\{x|\mu_{A}\left(x\right)\geq \alpha \right\}$, $B_{\alpha }=\left\{x|\mu_{B}\left(x\right)\geq \alpha \right\}$, where $A_{\alpha }$ is the α-cut of *A*, and $B_{\alpha }$ is the α-cut of *B*. It should be noted that the α-cuts of fuzzy numbers are intervals, thus it can be written that $A_{\alpha }=\left[a_{\alpha }^{-},a_{\alpha }^{+}\right]$ and $B_{\alpha }=\left[b_{\alpha }^{-},b_{\alpha }^{+}\right]$, where $a_{\alpha }^{-}<a_{\alpha }^{+}$, and $b_{\alpha }^{-}<b_{\alpha }^{+}$. On this basis, the probability of $A_{\alpha }$ being lower than $B_{\alpha }$ is defined as follows:

(2) $$P_{\alpha }\left(A_{\alpha }<B_{\alpha }\right)=\int_{a_{\alpha }^{-}}^{a_{\alpha }^{+}}\int_{max(a,b_{\alpha }^{-})}^{max(a,b_{\alpha }^{+})}\frac{1dadb}{\left(a_{\alpha }^{+}-a_{\alpha }^{-}\right)\left(b_{\alpha }^{+}-b_{\alpha }^{-}\right)}$$

**Figure 7:**
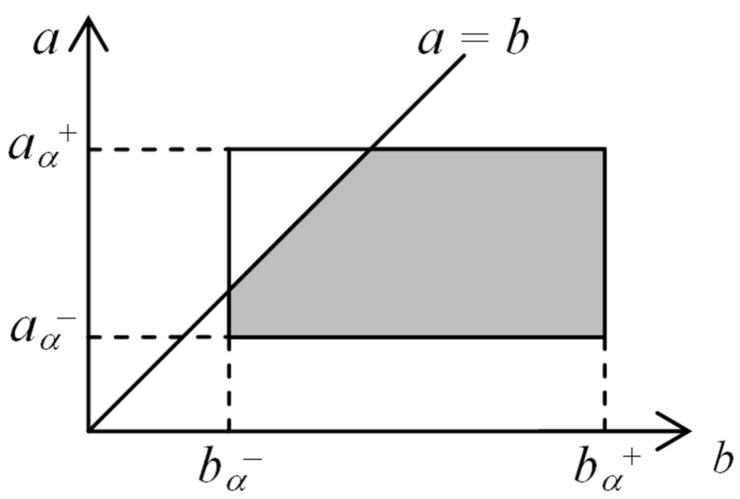
Geometrical interpretation of probabilistic α-cuts ordering.

A geometrical interpretation of the definition in Equation (2) is presented in Figure 7. It should be noted that value of the numerator in Equation (2) is equal to the area of the grey region in Figure 7, which includes such combinations of $a\in A_{\alpha }$ and $b\in B_{\alpha }$ that satisfy the condition a<b. The denominator in Equation (2) describes the area of the rectangle in Figure 7, which covers all possible combinations of $a\in A_{\alpha }$ and $b\in B_{\alpha }$.

Finally, the following formula is used to calculate the probability that fuzzy number *A* is lower than fuzzy number *B*:

(3) $$P\left(A<B\right)=\int_{0}^{1}\frac{\alpha \cdot P_{\alpha }\left(A_{\alpha }<B_{\alpha }\right)d\alpha }{0.5}.$$

As explained. above, in the proposed optimization procedure the fuzzy numbers represent values of objective function for candidate solutions. Let the fuzzy numbers *A* and *B* describe values of objective function for candidate solutions $x_{A}$ and $x_{B}$, respectively. The candidate solution $x_{A}$ is considered to be better than $x_{B}$ if P(A<B)>0.5.

## 4. Experimental Evaluation

The results of the experiments discussed in this section cover total vehicle delay, average speed, and travel time that were determined for the new proposed approach as well as for the existing decentralized traffic signal control methods from the literature [7,8,31]. Effectiveness of the traffic signal control was compared for the neuroevolution strategy applied to different neural network topologies, i.e., multi-layer perceptron (MLP), radial basis function artificial neural network (RBF ANN) and fully-connected neural network (FCNN). Moreover, the experiments were conducted using various implementations of the proposed approach, with different occupancy prediction procedures and different metaheuristic algorithms for synaptic weight optimization.

**Figure 8:**
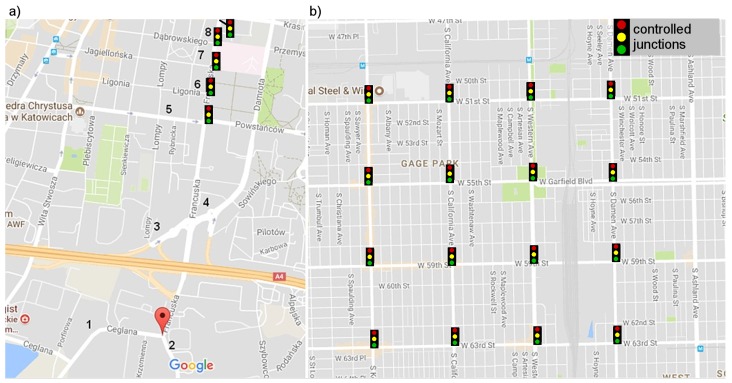
Test scenarios: (**a**) signalized arterial road; and (**b**) grid road network (based on google maps: http://maps.google.com).

**Figure 9:**
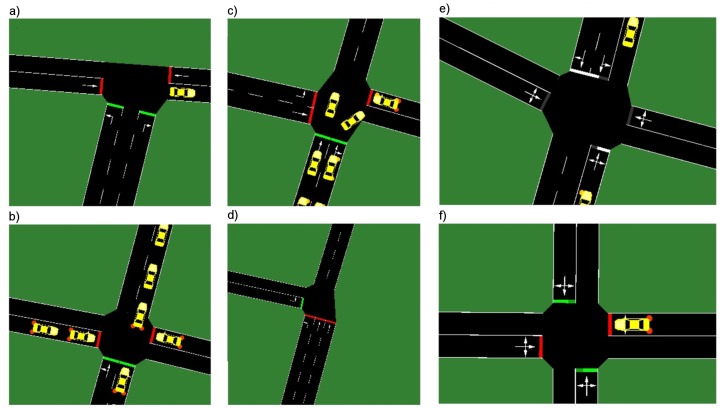
Intersections characteristic for: the first scenario (**a**–**d**); and the second scenario (**e**,**f**).

The proposed neuroevolution approach to decentralized traffic signal control was experimentally evaluated in two real-world scenarios. The first scenario takes into account a signalized artery (Francuska Street) with five intersections in the centre of Katowice, Poland. This is a one-way road with multiple inlets (Figure 8a), which often cause congestions. Each intersection has various number of lanes/geometry and thus different traffic light characteristics. In the second scenario (Figure 8b), the mesh road network of Gage Park district in Chicago was considered. Each of 16 simulated road intersections manages four-way traffic (one or two inbound and outbound lanes). To better illustrate the simulation scenarios, the intersections are presented in Figure 9. The intersections presented in Figure 9a–d are localized in Francuska Street (main traffic moves north), while intersections in Figure 9e,f are typical four-way intersections from the Gage Park district.

**Figure 10:**
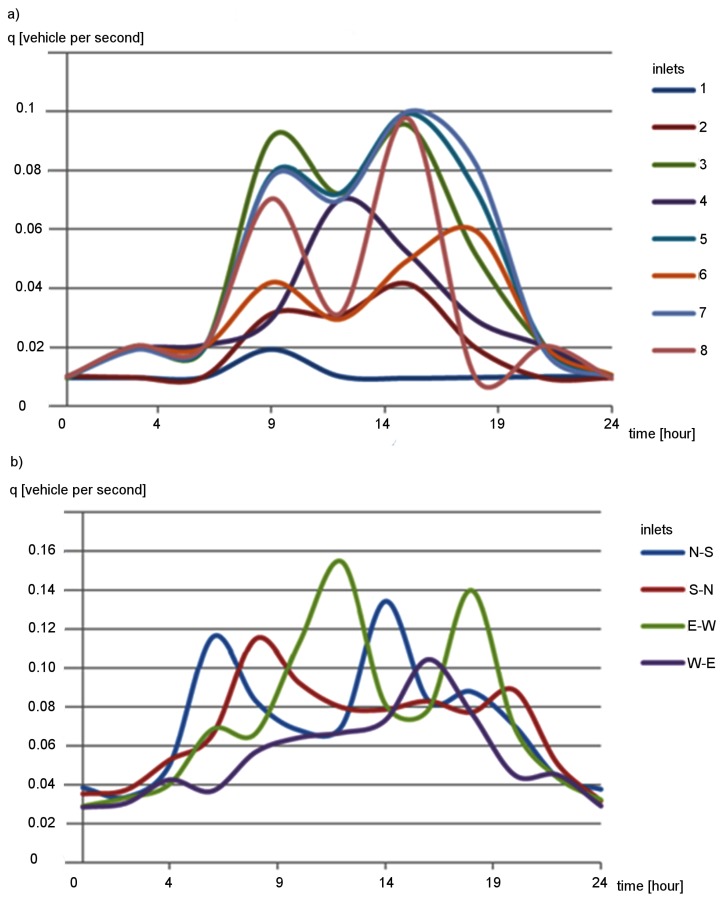
Profiles of traffic intensity (q): (**a**) arterial road scenario; and (**b**) grid road network scenario.

During simulations, the vehicles were randomly generated. The simulated traffic intensities were based on real-world daily traffic profiles (Figure 10) obtained from road-side sensor networks [32]. For the arterial road scenario, vehicles were inserted in inlets that are indicated in Figure 10a by numbers 1–8. The traffic intensity profiles for these inlets are shown in Figure 10a. In the case of the grid network scenario, the traffic intensity profiles, shown in Figure 10b, were assigned to particular traffic directions. For instance, the N–S profile was used to generate vehicles travelling from north to south. The vehicles were generated using the same intensity profile at each entrance of the road network for a given direction. It should also be noted that one simulation run corresponded to one day (24 h).

### 4.1. Prediction of Traffic Lane Occupancy

As i explained in Section 3, inputs of the proposed neural network ensemble include predicted future occupancies of traffic lanes ($d_{i}$). During experiments, two different prediction methods were implemented to evaluate the future occupancies: exponential smoothing [33] and k-nearest neighbors (k-NN) algorithm with data segmentation [34,35]. In the case of exponential smoothing, the future occupancy of lane *i* is calculated at time step *t* by using the following formula:

(4) $$d_{i}\left(t\right)=\epsilon \cdot \phi_{i}\left(t\right)+\left(1-\epsilon \right)\cdot d_{i}\left(t-1\right),$$

where ϵ∈[0,1] denotes smoothing constant, $d_{i}\left(t-1\right)$ is the occupancy of lane *i* predicted at previous time step, and $\phi_{i}\left(t\right)$ is current occupancy.

The occupancy of traffic lane *i* at time step *t* ($\phi_{i}\left(t\right)$) is determined as the quotient of the number of occupied cells and the total number of monitored cells in lane *i*. The monitored cells are those in which the vehicles can be detected. In this study, the number of monitored cells *n* for each traffic lane was set to 10, which corresponded to a distance of 75 m. This assumption enabled timely detection of vehicles approaching an intersection with maximum velocity (60 km/h). Moreover, it took into account the distances between intersection and the limited range of vehicle detection systems. Effectiveness of these settings was verified in preliminary experiments [36].

The second considered prediction method uses the k-NN algorithm with data segmentation. According to this method, the future occupancy of traffic lane is calculated as follows:

(5) $$d_{i}\left(t\right)=\sum_{g=1}^{k}\frac{\bar{\phi_{i}}\left(t_{g}+1,t_{g}+10\right)}{dist(t,t_{g})}/\sum_{g=1}^{k}\frac{1}{dist(t,t_{g})},$$

where $dist(t,t_{g})$ is Euclidean distance between state vectors representing traffic conditions observed at time steps *t* and $t_{g}$, *t* denotes current time step, $t_{g}$ (*g* = 1, 2, ..., *k*) are time points in historical data, where the nearest neighbors (historical state vectors most similar to the current state vector) have been found, and $\bar{\phi_{i}}\left(t_{g}+1,t_{g}+10\right)$ denotes average (historical) occupancy observed at time steps $t_{g}$ + 1,..., $t_{g}$ + 10. The state vector in this method consists of the traffic lane occupancies that were registered at a given time step (*t* or $t_{g}$) and at five previous time steps. Similarity between these vectors is determined using the Euclidean distance. At a given time point, the similar state vectors (nearest neighbors) are searched in selected segments of the historical data that were assigned to this specific time point. The selected segment corresponds to a time interval, which is expected to include traffic data that are similar with a given traffic state and provide accurate prediction. The data segments are determined in the pre-processing stage, prior to the k-NN prediction. Detailed information about the k-NN prediction algorithm and the segmentation procedure can be found in [35].

Effectiveness of the proposed approach was compared for three versions: without occupancy prediction, with occupancy prediction based on exponential smoothing, and with the k-NN occupancy prediction. In the case of the version without prediction, inputs $d_{i}$ were set to 0 and thus had no impact on the priorities calculated by the neural network ensemble.

**Figure 11:**
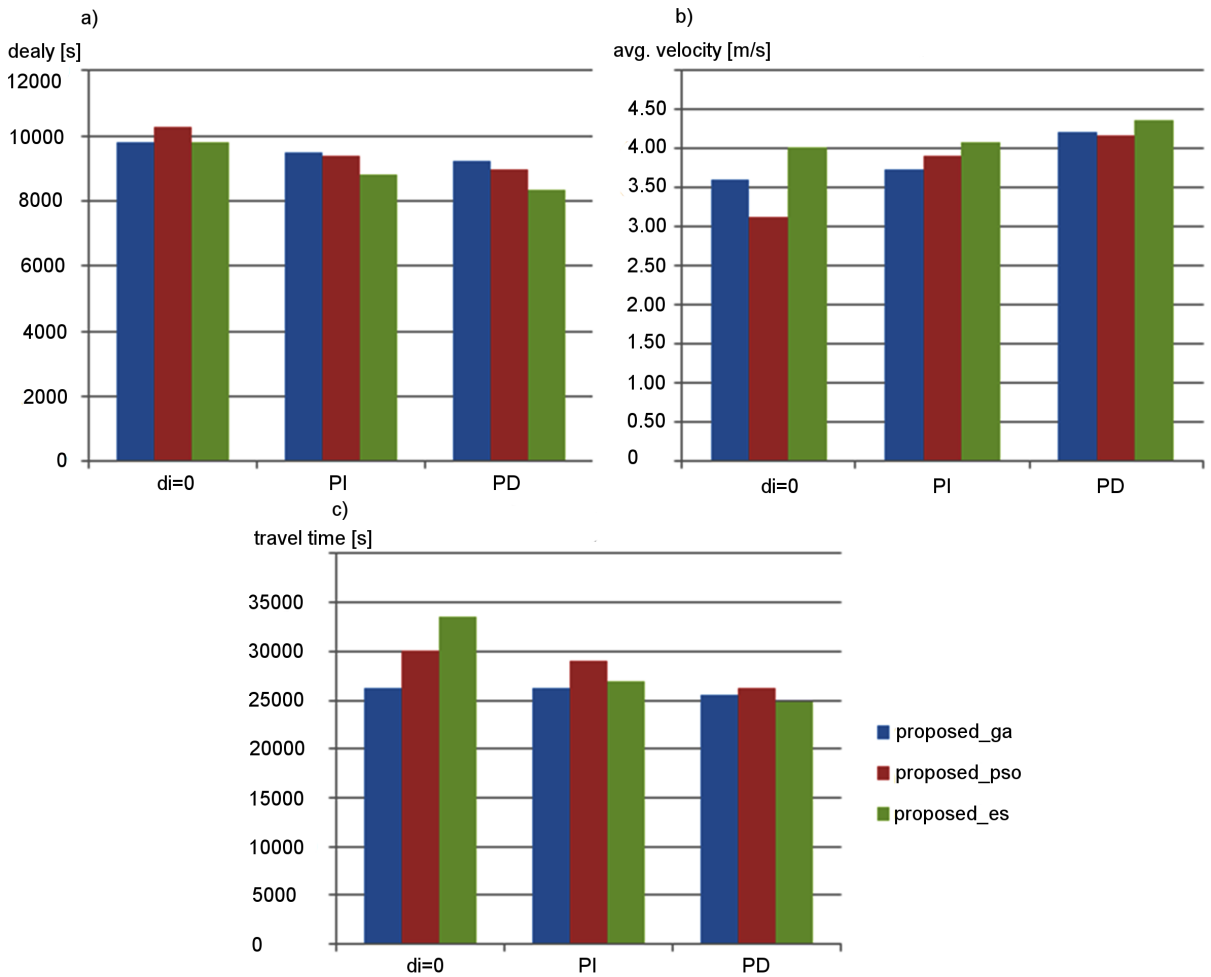
Effectiveness of proposed approach for different occupancy prediction methods: (**a**) delay; (**b**) average velocity; and (**c**) travel time.

Impact of occupancy prediction on effectiveness of the proposed approach was examined in simulation experiments. Results of these experiments for the signalized arterial road scenario are presented in Figure 11. They show that the utilization of the predicted lane occupancy improves the traffic control effectiveness. When using the simple exponential smoothing method (PI), it is possible to reduce delay, decrease travel time, and increase average velocity of vehicles by 20% on average. Optimal value of the smoothing constant (ϵ=0.15) was selected during preliminary experiments. An additional 10% increase in the traffic control effectiveness was achieved by using the more sophisticated prediction, which is based on k-NN algorithm (PD). Similar effects were observed for the second simulation scenario (the grid road network). Thus, the k-NN based prediction method was used to determine $d_{i}$ values in further experiments. It should be also noted that the parameters of the k-NN algorithm have been adjusted during preliminary tests to obtain the lowest vehicle delay. The suffixes _es, _ga, and _pso in the legend of Figure 11 indicate which metaheuristic algorithm was used to optimize the synaptic weights (evolutionary strategy, genetic algorithm, or particle swarm optimization, respectively).

### 4.2. Neural Network Topologies

During experiments, several neural networks with various topologies were used to evaluate priorities of signal groups for the decentralized traffic signal control. The following state-of-the-art topologies were considered: MLP [23], RBF ANN [37] and FCNN [38]. For these network topologies, the neuroevolution approach was implemented to optimize the synaptic weights.

**Figure 12:**
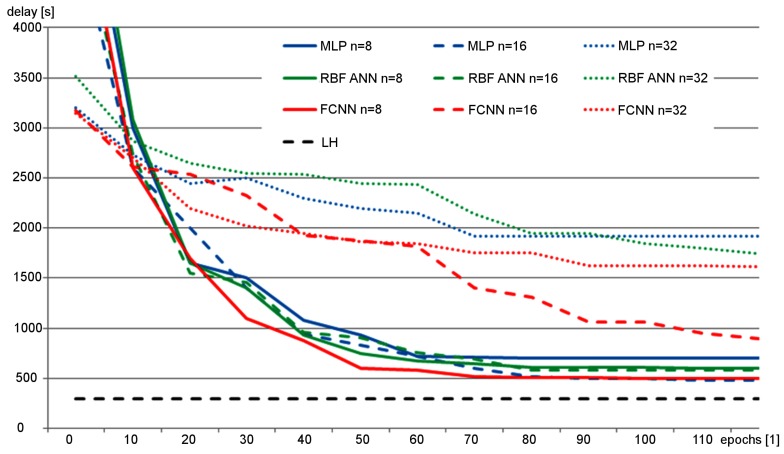
Vehicle delay observed during neuroevolution of ANNs with various topologies.

Figure 12 shows changes of vehicles delay during the neuroevolution process for the examined neural network topologies with different number of neurons (*o*). The results obtained for various neural networks (ANNs) are compared in Figure 12 with the delay achieved by the LH (Lammer and Helbing) algorithm [7], which is not based on ANNs. The compared ANNs included two hidden layers. The total number of neurons (*o*) was equally divided between those layers. It should be noted that for ANNs with one, three and four layers a lower effectiveness of the traffic control was observed than those achieved by the ANNs with two layers.

The vehicle delays in Figure 12 are average values calculated for the entire population of candidate solutions at given epoch. During this experiment, the neuroevolution was performed by using genetic algorithm to optimize the synaptic weights. Results of this research show that, due to significant number of synaptic weights, the complex ANNs with large number of neurons (o=32 for MLP and RBF ANN, o>16 for FCNN) could not be effectively evolved in reasonable time. On the other hand, the simple ANNs with lower number of neurons could not achieve the delay level of the standard LH algorithm. Nevertheless, the MLP and FCNN provided the best results for the small ANNs. Thus, several topologies of ANNs based on MLP and FCNN were considered during the next experiments.

**Figure 13:**
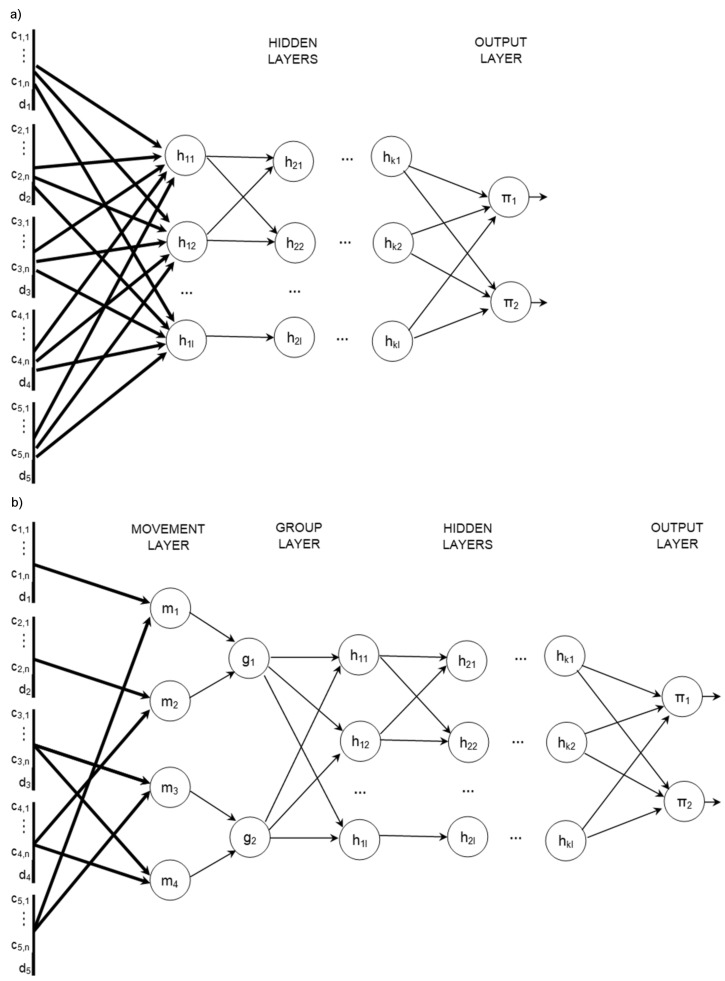
Topologies of the compared ANNs: (**a**) ANN_SW/DW, (**b**) ANN_DG.

Effectiveness of the traffic control was compared for several implementations of the decentralized algorithm that calculate the priorities of signal groups by using the proposed neural network ensemble and other ANNs with different topologies. The other considered ANNs topologies are presented in Figure 13. For these topologies, the number of hidden layers (*k*) and number of neurons (*l*) was modified to obtain the lowest vehicle delay. The ANN_DW/SW achieved the best results for *k* = 2 and *l* = 8. In the case of ANN_DW, the weights of connections between neurons in input layer and the first hidden layer are optimized independently for each traffic lane. ANN_SW uses the same weights vector for all traffic lanes. The ANN_DG topology includes the proposed data aggregation module with movement and group layers. In the case of ANN_DG, the weights of connections between input layer and movement layer are the same for all traffic lanes.

**Figure 14:**
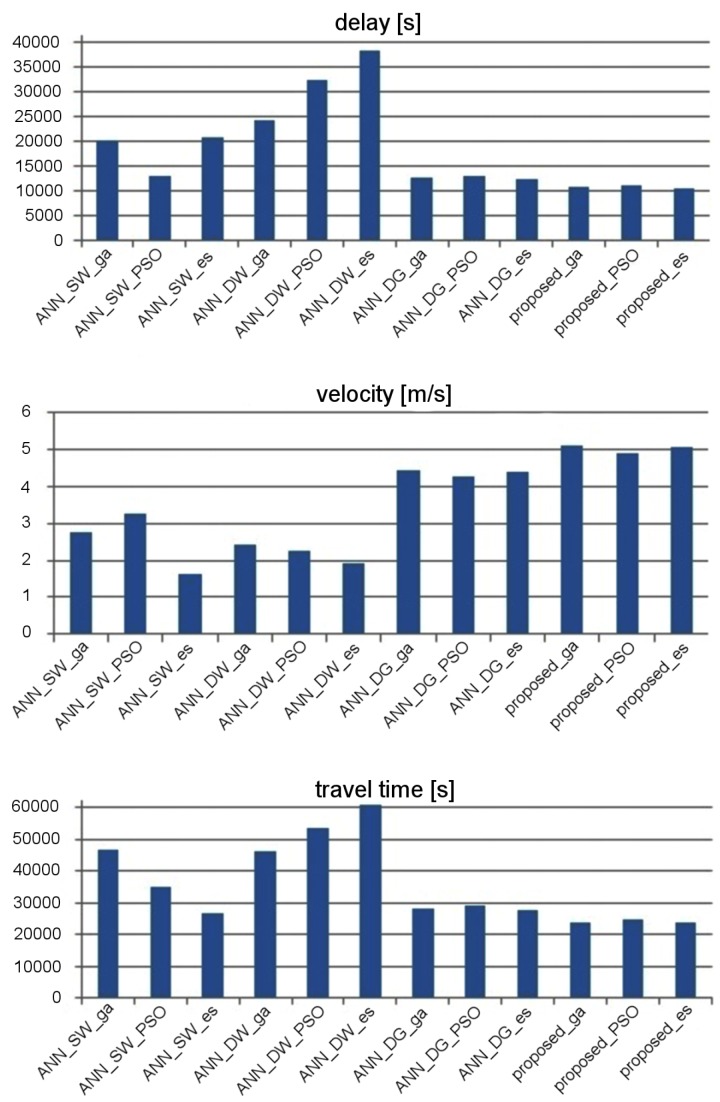
Effectiveness of traffic control for the considered ANNs in signalized arterial road.

**Figure 15:**
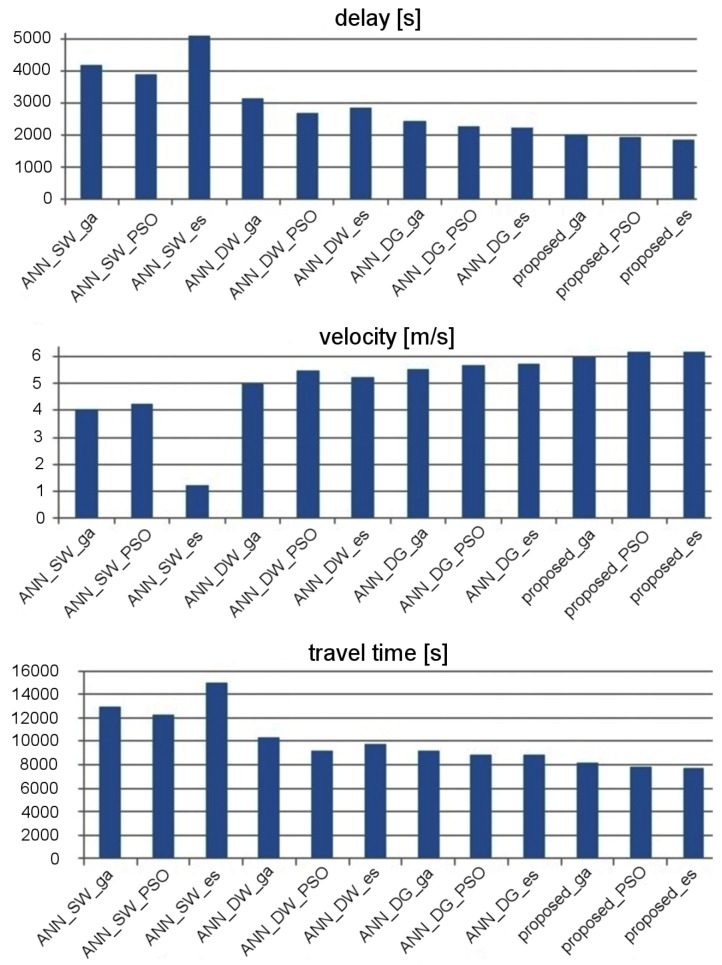
Effectiveness of traffic control for the considered ANNs in grid road network.

The effectiveness of traffic control was evaluated by taking into account stop delay, travel time, and average velocity of vehicles. Figure 14 and Figure 15 show the experimental results that describe effectiveness of traffic control for the considered ANN topologies. It should be kept in mind that the suffixes _es, _ga, and _pso in the labels of particular ANNs indicate which metaheuristic algorithm was used to optimize the synaptic weights. These results were averaged for 20 runs of simulation in SUMO [39]. It should be noted that the population-based metaheuristic methods, which are used in the proposed approach for synaptic weights optimization, introduce randomization of the candidate solutions at each step of the procedure (i.e., mutation in the case of the evolutionary strategy). Consequently, the final results of the metaheuristic optimization can be different after each execution of the algorithm. Therefore, the tests in SUMO were repeated 20 times to find an average result for each compared method. Based on these results, it can be observed that the effectiveness of traffic control can be improved by combining the proposed data aggregation module with standard ANN topologies. However, the best results were obtained (average speed increased by 11.2%, stop time decreased by 10.7%, and travel time decreased by 10.9% approximately to ANN_DG—second result) when using the proposed ensemble, which consists of the data aggregation module and the priority aggregation module with one FCNN for each signal group.

The results presented in Figure 14 show that ANN_SW enables more effective traffic control than ANN_DW in the case of the arterial road scenario. On the other hand, for the grid road network scenario (Figure 15), ANN_DW proved to be superior over ANN_SW. The network with additional movement and group layers (ANN_DG) gives better results than ANN_SW and ANN_DW for both scenarios. The best results in the considered scenarios were achieved by the proposed neural network ensemble, which includes the aggregation module with separate FCNN for each signal group.

**Figure 16:**
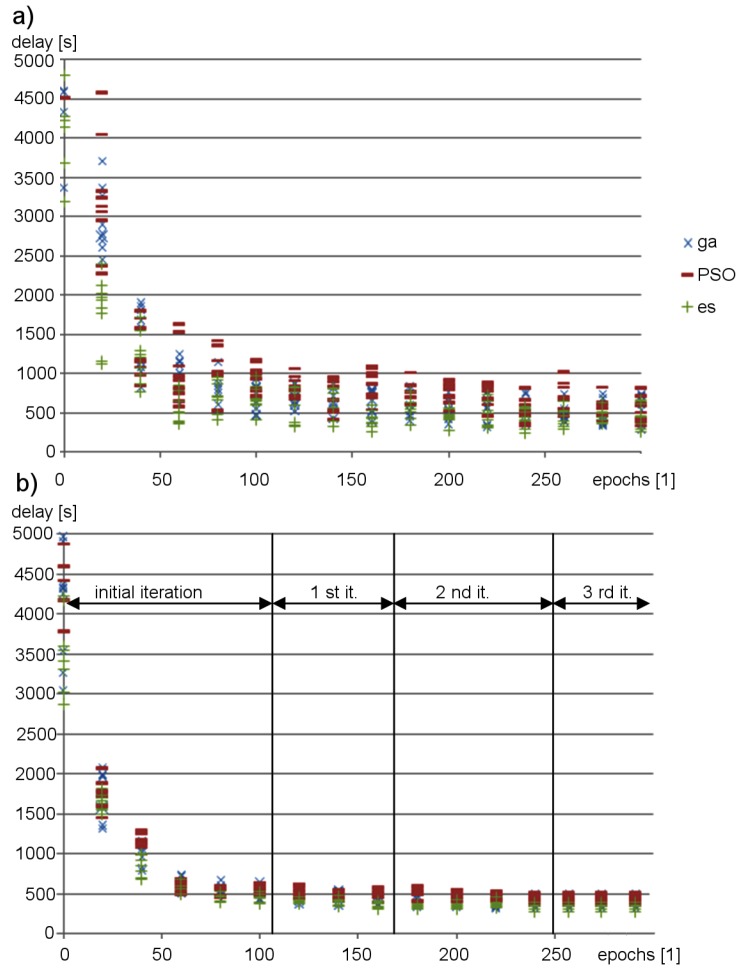
Changes of vehicle delay during optimization procedure: (**a**) ANN_DG; and (**b**) the proposed neural network ensemble.

The evolution of the proposed neural network ensemble is performed in iterations, as explained in Section 3.4. After initial optimization of the synaptic weights, successive iterations were performed to adjust the topology and synaptic weights only for the priority evaluation module. Figure 16 compares results of the proposed neuroevolution approach with those obtained during optimization of the synaptic weights for ANN_DG, based on the standard metaheuristic algorithms, without iterative modifications of the neural network topology. The scatter plots in Figure 16 show vehicle delays achieved during subsequent epochs for 10 runs of the optimization procedures. It can be observed in these results that for the proposed neuroevolution strategy the obtained vehicle delay is close to the minimum in each optimization run. In the case of ANN_DG, the delay values are spread over a wide range, which means that execution of the optimization procedure can give unsatisfactory results. The proposed approach enables finding the optimal solution in shorter time. One of the reasons behind this result is the fact that in the case of the proposed FCNNs ensemble a higher effectiveness of traffic control can be obtained using a lower number of neurons in comparison to other ANN architectures. The ANN_DG needs 16 neurons on average in hidden layers to achieve good results. For the proposed method, this number is reduced by half.

### 4.3. Comparison with State-of-the-Art Decentralized Traffic Control Strategies

**Figure 17:**
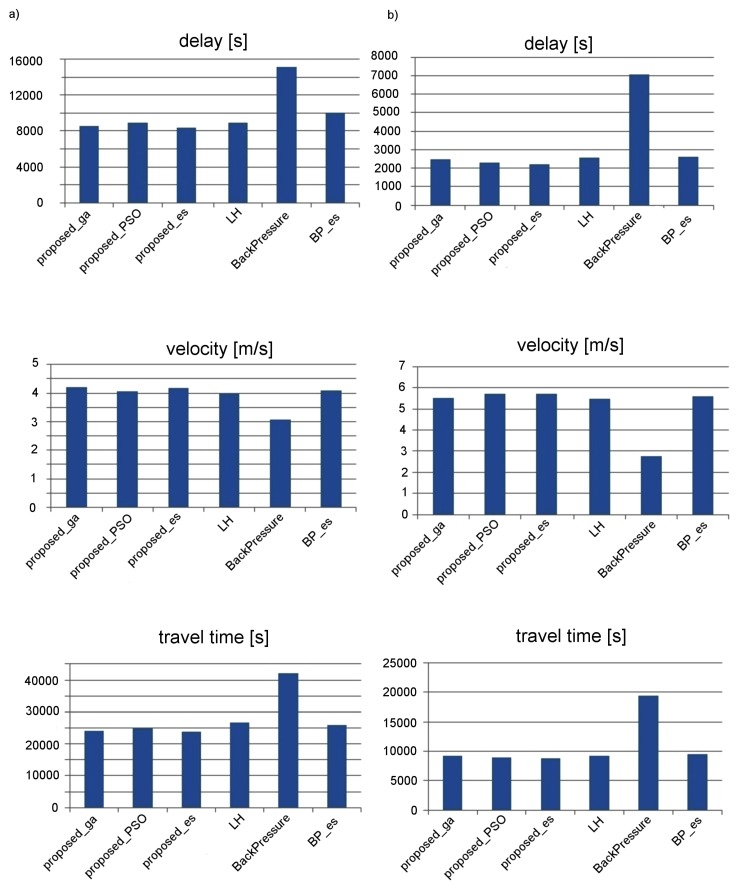
Effectiveness of traffic control for the compared methods: (**a**) signalized arterial road; and (**b**) grid road network.

The effectiveness of the proposed neuroevolution-based traffic signal control strategy was compared with the existing decentralized traffic signal control methods from the literature. Three different state-of-the-art methods were taken into account in this study: the method LH proposed by Lammer and Helbing [7], the back-pressure algorithm [31] and the method based on self-adaptive evolutionary strategy BP_es [8]. The performance indicators, which were used for evaluation of the above mentioned methods, include vehicle delay, average speed, and travel time (Figure 17). The results shown in Figure 17 were obtained as average of 20 simulation runs for both scenarios (signalized arterial road and grid road network). Various versions of the proposed approach were taken into consideration. As explained above, the suffixes _es, _ga, and _pso indicate which metaheuristic algorithm was used to optimize the synaptic weights in a given version of the proposed method.

The proposed approach proved to give superior results compared to the state-of-the-art methods. When comparing the proposed approach with the back pressure method, it can be observed that the traffic delay was reduced by 40%. In the case of the more sophisticated methods (LH and BP_es), the decrease of delay is also significant (equal to 7% and 4%, respectively). The performance of the proposed approach depends on the type of the metaheuristic algorithm, which is used to optimize the synaptic weights during neuroevolution. The best results were achieved by using the evolutionary strategy and the genetic algorithm. Slightly worse results were obtained for the PSO algorithm.

The results presented in this section describe the overall effectiveness of traffic signal control for entire road network with multiple intersections. However, detailed results obtained for particular intersections also confirm the above discussed advantages of the proposed approach. The introduced neural network ensemble and neuroevolution strategy enable appropriate evaluation of signal group priorities for the decentralized traffic control at various intersections.

## 5. Conclusions

In this paper, a new neural network ensemble is proposed, which consists of data an aggregation module and a priority evaluation module. The priority evaluation is necessary for implementation of the decentralized traffic control strategy, which uses the priorities to decide which traffic streams at an intersection should get the green signal. The proposed ensemble can be used with various sensor networks, i.e., road side sensors or visual detection sensors, providing their data are represented as states (occupancies) of cells. The novelty of the proposed approach lies in both the design of the ANN ensemble and the neuroevolution strategy, which was introduced to optimize topology and synaptic weights of the ANNs included in the ensemble.

The decentralized traffic signal control system, based on the proposed ANN ensemble and neuroevolution strategy, was experimentally evaluated in real-world scenarios. Advantages of the proposed approach were confirmed during extensive simulation experiments. The proposed solution was compared against state-of-the-art decentralized traffic control algorithms. The experiments showed that the proposed solution can achieve better results than the state-of-the-art methods in terms of reduced vehicle delay, shorter travel time, and increased average velocity. The experimental results also confirmed that a higher performance of traffic signal control can be achieved when using the predicted occupancies of traffic lanes as additional input data for the ANN ensemble. The level of performance improvement strongly depends on accuracy of the prediction method.

It should also be noted that the sensing technologies, which collect information about number of vehicles on the road, would be insufficient to achieve the high performance of traffic signal control. During research, it was observed that information regarding locations of vehicles was necessary to improve the performance of signal control. This fact was confirmed by the worse results of the back-pressure algorithm. In the proposed approach, the locations of vehicles are represented by determining occupied cells of traffic lanes. This approach could be further improved by using the sensor network optimization method proposed in [2] to decrease the number of monitored cells.

The proposed approach is suitable for applications based on mobile edge computing (MEC) and fifth-generation mobile communication technology (5G) [40,41]. In such applications, the vehicle localization data can be collected from mobile devices via 5G network. Priorities of traffic streams can be evaluated by using the proposed neural network ensemble at MEC servers and reported to local traffic controllers at intersections.

Future research directions will be related to different neuroevolution strategies that could enable on-line tuning of ANN parameters during operation of the traffic control system. Another interesting topic for future investigations is introduction of dynamic synaptic weight changes during the traffic control procedure. Finally, various traffic prediction methods for input data determination in the proposed ensemble can be tested.
